# Morphological characteristics and abundance of prokaryotes associated with gills in mangrove brachyuran crabs living along a tidal gradient

**DOI:** 10.1371/journal.pone.0266977

**Published:** 2022-04-14

**Authors:** Elisa Garuglieri, Jenny Marie Booth, Marco Fusi, Xinyuan Yang, Ramona Marasco, Tumeka Mbobo, Emanuela Clementi, Luciano Sacchi, Daniele Daffonchio

**Affiliations:** 1 Biological and Environmental Sciences and Engineering Division (BESE), King Abdullah University of Science and Technology (KAUST), Red Sea Research Center, Thuwal, Saudi Arabia; 2 Joint Nature Conservation Committee, Peterborough, United Kingdom; 3 National Research Foundation-South African Institute for Aquatic Biodiversity Institute, Makhanda, South Africa; 4 South African National Biodiversity Institute, Kirstenbosch Research Centre, Cape Town, South Africa; 5 Department of Botany and Zoology, Centre for Invasion Biology, Stellenbosch University, Stellenbosch, South Africa; 6 Dipartimento di Biologia e Biotecnologie “L. Spallanzani”, Università di Pavia, Pavia, Italy; Free University of Bozen-Bolzano, ITALY

## Abstract

Due to the chemico-physical differences between air and water, the transition from aquatic life to the land poses several challenges for animal evolution, necessitating morphological, physiological and behavioural adaptations. Microbial symbiosis is known to have played an important role in eukaryote evolution, favouring host adaptation under changing environmental conditions. We selected mangrove brachyuran crabs as a model group to investigate the prokaryotes associated with the gill of crabs dwelling at different tidal levels (subtidal, intertidal and supratidal). In these animals, the gill undergoes a high selective pressure, finely regulating multiple physiological functions during both animal submersion under and emersion from the periodical tidal events. We hypothesize that similarly to other marine animals, the gills of tidal crabs are consistently colonized by prokaryotes that may quantitatively change along the environmental gradient driven by the tides. Using electron microscopy techniques, we found a thick layer of prokaryotes over the gill surfaces of all of 12 crab species from the mangrove forests of Saudi Arabia, Kenya and South Africa. We consistently observed two distinct morphotypes (rod- and spherical-shaped), positioned horizontally and/or perpendicularly to the gill surface. The presence of replicating cells indicated that the prokaryote layer is actively growing on the gill surface. Quantitative analysis of scanning electron microscopy images and the quantification of the bacterial 16S rRNA gene by qPCR revealed a higher specific abundance of prokaryote cells per gill surface area in the subtidal species than those living in the supratidal zone. Our results revealed a correlation between prokaryote colonization of the gill surfaces and the host lifestyle. This finding indicates a possible role of prokaryote partnership within the crab gills, with potential effects on animal adaptation to different levels of the intertidal gradient present in the mangrove ecosystem.

## Introduction

The colonization of land habitats by aquatic organisms is one of the most important events in the history of life on Earth [1–3]. Due to the chemico-physical differences between air and water (i.e., higher viscosity, thermal capacity, and lower oxygen content in water), the transition from marine to terrestrial life poses several eco-physiological challenges to living beings [4]. Desiccation is a main risk on land and, consequently, organisms must finely regulate their osmolarity and respiration to survive; moreover, environmental variability requires adjustments of life cycles, sensory reception and stress responses [4].

Recent studies have demonstrated that plants were the first multicellular organisms to conquer the terrestrial ecosystem with the help of mycorrhizal fungi [5] and bacterial symbionts [6]. Although these results shed light on the role of symbiosis in the adaptation of plants to terrestrial habitats, few studies have investigated the metazoan counterpart [7–9]. Arthropods show very ancient and complex adaptative radiations to life on land, recurring multiple times in different geological periods [3,10]. They have evolved morphological, physiological and behavioural adaptations, such as cement and wax layers of the epicuticle to reduce water loss, the evolution of tracheae for air-breathing and haemoglobin/hemocyanin as a storage/respiratory pigment for oxygen transportation [11]. Brachyuran crabs are among the best examples of these adaptations, showing a wide colonization range of both marine and terrestrial habitats, with a remarkably high number of species inhabiting tidal ecosystems [12,13]. This adaptive flexibility, supported by paleontological evidence [3,14,15], indicates that brachyurans are still undergoing the process of terrestrial adaptation, making them a representative group for the study of this ongoing process [12,16].

Among tidal ecosystems, mangrove forests harbour rich and highly specialized ecological communities mainly shaped by the tidal environmental gradients [17–19]. A high number of brachyuran crab species are found in mangroves, showing high metabolic flexibility and lifestyles adapted to different tidal habitats, ranging from shallow subtidal to supratidal environments [12,20]. In these crabs, the gill is one of the main organs involved in the adaptation to the terrestrial environment [21]. Gills, constituted by arrays of plate-like structures called lamellae, are primarily responsible for gas exchange, but they are also involved in osmoregulation, pH regulation and nitrogen catabolite excretion [20]. Enclosed within the gill chamber, the gill microenvironment is characterized by an abundance of nutrients, such as carbon and nitrogen catabolites [20], suitable to support microbial metabolisms; for this reason, lamellae are a perfect niche for microorganisms to adhere and thrive. Although an exhaustive number of studies have described the morphology and physiology of the gill as an organ, the study of the microbial component associated with the gill as a micro-environment has been overlooked [22,23]. Due to their high metabolic plasticity, adaptability and functional redundancy [24], microbial communities can offer important advantages to the host during the colonization process of terrestrial habitats [8,25–31]. In such a process, morphology, topology, density and attachment modes of microbes can unravel both their physiological and mechanical relationships with their ecological niche [32,33].

Here, we hypothesize that brachyuran crab species living across the mangrove tidal gradient (i.e., subtidal, intertidal and supratidal zones) may host prokaryotic communities on their gills characterized by differential abundance, cell morphology and topological patterns of distribution, in accordance with the tidal level they colonize. This may witness an evolutionary partnership between the crab and its microbiota leading to (i) a selective adaptation of prokaryotes as a result of the host lifestyle (subtidal, intertidal and supratidal) and (ii) a possible role of microorganisms in the adaptation of the host to the environment, as it has occurred in the terrestrialisation of plants [5,34,35].

## Methods

### Ethical statement

All experiments described in the manuscript were conducted in accordance with the Guidelines for the Treatment of Animals in Behavioral Research and Teaching from the Animal Behavior Society. Animals used in our experiments were maintained and treated in compliance with the guidelines specified by King Abdullah University of Science and Technology. In addition, all necessary permits were in hand when the research was conducted and all experiments and procedures were approved by the King Abdullah University of Science and Technology. Prior to sampling, we obtained all the permits by the local authorities.

### Species selection, identification and sampling

The present study is focused on intertidal crabs, as a group challenged by emersion/submersion events [20]. Sampled crab species, their geographical origin, habitat and number of specimens per each analytical technique are reported in Table 1. A total of 12 intertidal mangrove crab species were considered and subdivided into three different categories based on the tidal level they occupy: (i) subtidal, mainly dwelling in the shallow coastal zone (never emerging from water), (ii) intertidal, mainly dwelling in the intertidal zone (experiencing daily submersion and emersion), (iii) supratidal, mainly dwelling in the higher intertidal habitat (rarely being submerged by water). One subtidal species (*Thalamita crenata* [Ruppell, 1830]) and one supratidal species (*Ocypode saratan* [Forskal, 1775]) were chosen to represent crabs adapted to the two extremes of the tidal gradient, while the remaining ten species were selected from the intertidal zone. Crabs from the intertidal zone were further sub-divided into four groups based on their taxonomy: Grapsidae (2 species: *Metopograpsus oceanicus* [Hombron & Jacquinot, 1846] and *Metopograpsus messor* [Forskal, 1775]), Sesarmidae (3 species: *Neosarmatium africanum* [Ragionieri, Fratini & Schubart, 2012], *Parasesarma guttatum* [A. Milne-Edwards, 1869] and *Parasesarma catenatum* [Ortmann, 1897]), Macrophtalmidae (1 species: *Macrophthalmus depressus* [Ruppell, 1830]) and Gelasiminae (4 species: *Tubuca urvillei* p H. Milne Edwards, 1852], *Paraleptuca cholorophthalmus* [H. Milne Edwards, 1837], *Cranuca inversa* [Hoffmann, 1874] and *Austruca albimana* [Kossmann, 1877]).

**Table 1:** Sampling details: Location, tidal level occupied and the number of specimens collected for each analysis are shown for each species.

Species	Country	Location	Tidal level	Number of individuals		
				SEM imaging	SEM counting	qPCR
*Thalamita crenata*	South Africa,	Mngazana	Subtidal	10	10	10
	Saudi Arabia	Makkah	Subtidal	10	10	10
*Parasesarma catenatum*	South Africa	Mngazana	Intertidal	10	6	-
*Parasesarma guttatum*	Kenya	Gazi Bay	Intertidal	5	5	-
	Saudi Arabia	Farasan		5	5	-
*Paraleptuca chlorophthalmus*	South Africa	Mngazana	Intertidal	10	10	-
*Neosarmatium africanum*	South Africa	Mngazana	Intertidal	10	8	-
*Metopograpsus oceanicus*	Saudi Arabia	Makkah	Intertidal	10	8	-
*Metopograpsus messor*	Saudi Arabia	Makkah	Intertidal	10	8	10
*Austruca albimana*	Saudi Arabia	Makkah	Intertidal	10	8	10
*Tubuca urvillei*	Kenya	Gazi Bay	Intertidal	5	-	-
	Saudi Arabia	Farasan		5	-	-
*Cranuca inversa*	Saudi Arabia	Makkah	Intertidal	5	5	10
		Farasan		5	5	-
*Macrophthalmus depressus*	Saudi Arabia	Makkah	Intertidal	10	-	-
*Ocypode saratan*	Saudi Arabia	Makkah	Supratidal	10	8	10

SEM imaging refers to the SEM micrographs taken to study the gill morphology, SEM counting refers to the images selected for cell counting (see Methods section for further details).

Four mangrove forests were chosen for crab collection: Farasan Island (Jizan district, Saudi Arabia; 16°477’N, 42°39’E), KAUST Ibn Sina Research Station (Makkah district, Saudi Arabia; 22°20’N, 39°05’E), Gazi Bay (Kwale district, Kenya; 4°22’S, 39°30’E), and Mngazana (Eastern Cape province, South Africa; 31°42’S, 29°25’E).

Regional identification guides, as well as local and regional species inventories and online resources such as OBIS (www.obis.org) for biogeography and World Register of Marine Species (WoRMS) [36] for taxonomy, were used to identify the species.

Adult male specimens were collected from each site, animals were dissected under sterile conditions and gills were extracted within 30 min of collection. Different storage methods were used for different subsequent microscopy and quantitative molecular analyses.

### Sample preparation for scanning electron microscopy (SEM) and transmission electron microscopy (TEM)

Scanning and transmission electron microscopy techniques (SEM and TEM, respectively) were used to assess the presence and capture fine-scale morphological details of microorganisms colonizing the gill surfaces in the 12 collected species (Table 1). The prokaryotic physical interactions with the host tissue were also investigated. For conventional SEM, each dissected gill was rinsed three times for 15 min with 0.1 M sodium cacodylate buffer (pH 7.3), fixed in the same buffer containing 1% osmium tetraoxide (OsO_4_) and stored at 4°C until further microscopic analyses. Following a triple rinse with distilled water, the fixed gill samples were dehydrated in an ascending series of ethanol dilutions (30%, 50%, 70%, 90%; 15 min each) and rinsed twice for 15 min in absolute ethanol. Samples were then transferred into absolute ethanol solutions with increasing concentrations of hexamethyldisilazane (HMDS; 33%, 50%, 66%; 20 min in each solution), then resuspended in absolute HMDS and left to evaporate overnight. For large samples, HMDS was evaporated using a critical point dryer (Autosamdri®-815B, Tousimis, Rockville, USA). Samples were then stub-mounted on adhesive carbon tape and sputter-coated with a 5 nm layer of Au/Pb using a K575X sputter coater (Quorum Technologies, Laughton, UK). Samples were observed and imaged with a TENEO FEI SEM, Magellan FEI SEM or Quanta 600 FEI SEM (FEI, Hillsboro, USA). For TEM, the gill sections (80 nm) were prepared as previously described by Sacchiand colleagues [37], and examined under a Titan FEI TEM (FEI, Hillsboro, USA) at 200 keV acceleration voltage. For CRYOSEM, samples were washed in 0.1 M cacodylate buffer and rinsed in distilled water before imaging with a Nova Nano SEM (Thermo Fisher Scientific, Waltham, USA) equipped with a CRYO stage (Quorum Technologies, Laughton, UK). All SEM imaging was performed in the Imaging and Characterization Core Lab (KAUST) and TEM imaging at the Department of Biology and Biotechnology of the University of Pavia (Italy).

### SEM image processing

SEM images were obtained from ten crab species (Table 1) to count the prokaryotic cells adhering to the crab gill surfaces. Notably, *T*. *urvillei* and *M*. *depressus* were discarded due to the lack of suitable pictures for the counting method requirements (minimum of 5 pictures with adequate magnification and orthogonal observation of a flat surface). For each species, five to ten individuals were chosen and for each individual, a single SEM image of the gill surface was selected to perform the quantification (Table 1). Using the free software ImageJ 1.52a [38], a 225 μm^2^ grid was overlayed on the original image (only in cases where the gill surface was flat and orthogonally imaged.) Three grids were counted for each image and only prokaryotic cells with a clear attachment to the gill surface within the grid borders were considered. Counting was performed by software automatic recognition and then manually checked to maximize accuracy. Prokaryote cells were classified by their morphology (i.e., rod-shaped, spherical and spiral), surface appearance (i.e., rough and smooth) and relative orientation/adhesion mode with respect to the gill surface (horizontal and perpendicular to the gill tissue). The total microbial density and counts of different morphologies, appearance and adhesion were normalized per mm^2^ of gill surface for each species.

### Total DNA extraction and quantification of prokaryote communities by quantitative PCR (qPCR)

For the quantification of gill bacteria via quantitative PCR amplification of the 16S rRNA gene, a selection of five species covering the entire tidal gradient (Table 1) was used: *T*. *crenata*, *M*. *messor*, *A*. *albimana*, *C*. *inversa* and *O*. *saratan*. Ten adult male specimens were collected from the Saudi Arabia site (Table 1) and the sampled gills were stored in RNAlater (Sigma-Aldrich, USA) stabilization solution at –20°C. Before analysis, crab gills were thawed at room temperature, gently rinsed with sterile PBS three times to remove RNAlater reagent, then homogenized manually with PBTP pestles (VWR, Radnor, USA) in 1.5 mL sterile tubes. The total DNA extraction of crab gills was performed using 50 mg of gill tissue and a DNeasy Blood and Tissue Kit (Qiagen, Hilden, Germany) following the manufacturer’s protocol. Absolute abundances of the number of copies of the bacterial 16S rRNA gene were determined using the Eub338/Eub518 primer-set (specific for eubacteria Domain) as described by Frierer and colleagues [39] and Callegari and colleagues [40]. To obtain the standard gene of interest, the fragment was amplified from environmental DNA (size ± 180 bp). PCR products were purified with the Wizard® SV Gel and PCR Clean-Up System (Promega Madison, USA) and ligated to vectors pCRTM 2.1-TOPO®, then cloned into TOP10 *Escherichia coli* competent cells (TOPO® TA Cloning® Kit, Thermo Fischer Scientific, Waltham, USA). Cultures of the transformant *E*. *coli* were incubated overnight on LB agarose medium. Correctly transformed colonies were isolated with the Pure Yield Plasmid Miniprep (Promega, Madison, USA) and used as a template to amplify the region of insertion of the fragment of interest with the primer-set M13F/M13R. PCR products were purified, quantified with the Qubit dsDNA HS Assay Kit (Thermo Fisher Scientific, Waltham, USA). Serial dilutions of the standards were prepared and quantified using the Qubit dsDNA HS Assay Kit, and standard curves were constructed with a series of dilutions ranging from 50 to 5 × 10^7^ copies of PCR product per microliter. Each sample was diluted to 1 ng/μL to be used as template DNA. Samples with very low concentrations were used undiluted. One sample was chosen as an inter-run calibrator, quantified in all qPCR experiments to normalize the results of different runs. Reaction mixes were prepared with the Platinum^®^ SYBR^®^ Green qPCR SuperMix UDG (Invitrogen, Waltham, USA) following the manufacturer’s indications. The volume of the reaction mix was 50 μL, containing 25 μL Platinum^®^ SYBR^®^ Green qPCR SuperMix UDG, 0.3 μL ROX reference dye, 1 μL of each primer (10 μM), 2 μL template DNA (2 ng) and 20.7 μL ddH_2_O. PCR conditions were the following: 95°C for 2 min followed by 40 cycles at 95°C for 15 s and 60°C for 15 s, and a melt curve analysis was constructed by increasing the temperature from 60°C to 95°C. PCR was run in a QuantStudio3 Real-Time PCR System (Thermo Fisher Scientific, Waltham, USA), final results were analyzed with the dedicated software QuantStudio™ Design & Analysis Software. All the standards and the samples were run in triplicate with *R*^2^ between 0.99870 and 0.99980 and amplification efficiencies between 93% and 98%. To estimate bacterial abundance, the absolute values of the 16S rRNA gene were divided by a mean of 4 gene copies/bacterial cell-based on literature [40–45] and calculated through the Ribosomal RNA Database (rrnDB, Lee ZM-P: http://rrndb.mmg.msu.edu/search.php) based on the most abundant phyla associated to crab gills: Actinobacteria, Proteobacteria and Bacteroidetes [23,46]. Values were then normalized per mg of gill fresh weight.

### Statistical analysis

All statistical analyses were performed using R software (version 3.6.1, R Core Team, 2020).

For analyses based on SEM images, the total abundance of prokaryotes (response variable) among crab tidal adaptation levels was tested (explanatory variable with the levels: subtidal species, intertidal Sesarmidae, intertidal Grapsidae, intertidal Gelasiminae species and supratidal species). Data were tested for uniformity of variance and normal distribution before ANOVA analysis (Type III for unbalanced datasets); pairwise comparisons were analysed using Tukey post hoc tests. We used multivariate generalized linear model analysis (GLM) (R, package mvabund [47]) to test differences in prokaryotic morphology abundance (response variable) among different intertidal levels (explanatory variable with the levels: subtidal species, intertidal Sesarmidae, intertidal Grapsidae, intertidal Gelasiminae species and supratidal species) as explanatory variables. To explore the discriminant morphology along the tidal gradient, we performed a univariate GLM using mvabund.

Differences in specific prokaryotic morphological combinations among crab tidal adaptation levels were analysed using a negative bimodal fit model glm.nb() with the R MASS package [48]. Pairwise analyses were performed with the emmeans package in R [49].

Quantitative PCR data of absolute bacterial abundance (normalized per mg of gill fresh weight) were tested for uniformity of variance and normal distribution before ANOVA analysis among crab species living at different levels of tidal adaptation (explanatory variable with the levels: subtidal species, intertidal species and supratidal species); pairwise comparisons were analysed using Tukey post hoc tests.

## Results

### Gill microbial coverage and prokaryotic morphology

**Fig 1:**
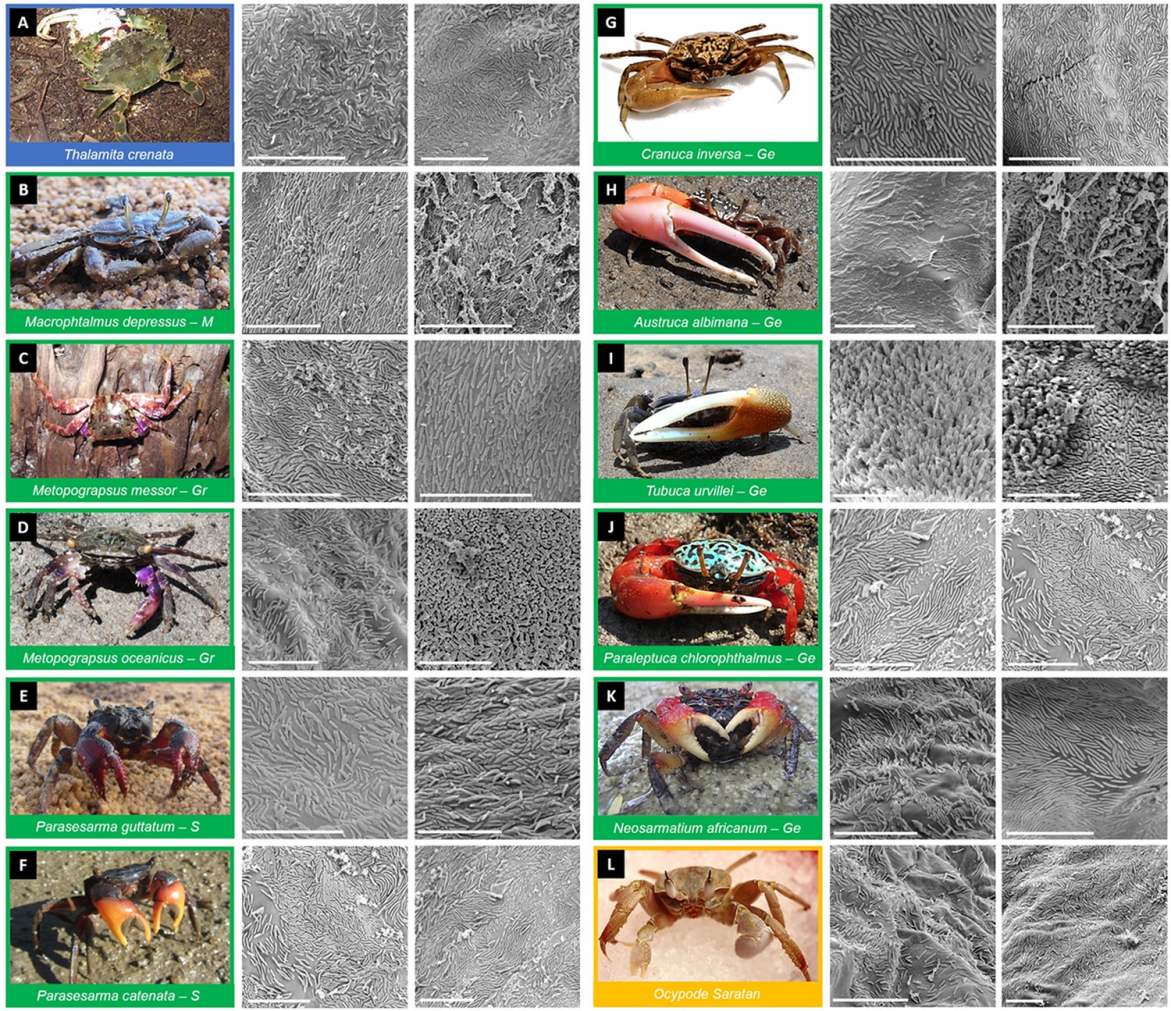
Prokaryotic colonization imaging of crab gills. Two representative SEM images of the microbial communities associated with gills are shown for each of the 12 crab species studied (**A**–**L**). The colours indicate the intertidal level inhabited: Blue subtidal, green = intertidal, yellow = supratidal. Letters in intertidal species show define each taxon-related intertidal group: Gr = Grapsidae, Ge = Gelasminidae, M = Macrophtalmidae, S = Sesarmidae. Scale bar corresponds to 10 μm (photo credit and copyright: Marco Fusi **A-F**, **H-K,** and Elisa Garuglieri **G, L**. Marco Fusi and Elisa Garuglieri publish these pictures under a CC BY licence).

**Fig 2:**
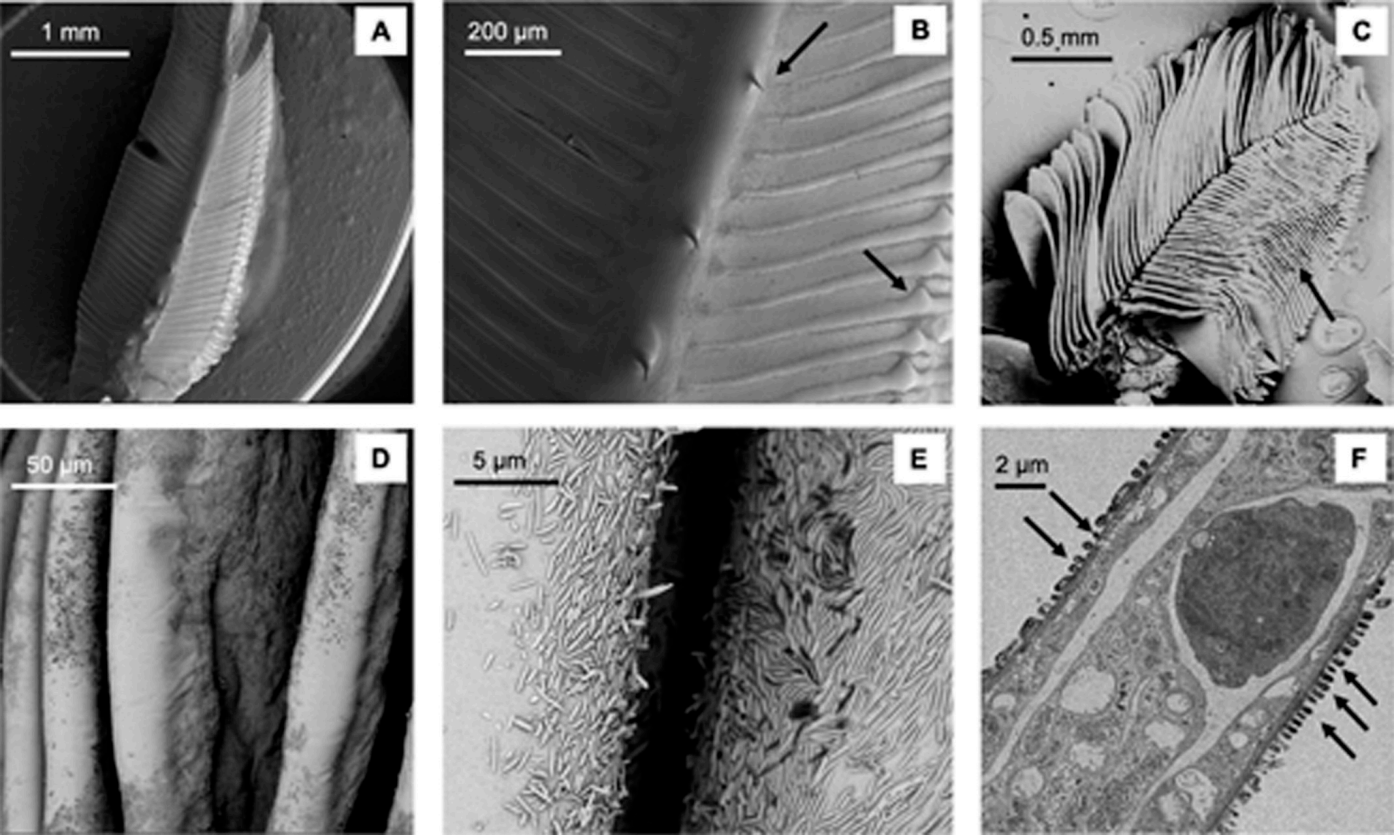
Gill structure morphology in the crab species *Cranuca inversa*. Image of a single gill observed with cryo-SEM at low magnification (**A**). Higher magnification showing gill lamellae connected by a membrane-like structure, black arrows indicate the preservation of the spine and bulb-like structures (**B**). Overview of the gill structure and lamellae organisation and the arrow shows the thicker part of the lamellae that prevent the collapse of the structure during emersion (**C**). Image of a single gill observed with conventional SEM at low magnification (**D**). Magnification of panel **D** showing separate gill lamellae covered by dark rough patches corresponding to bacterial coverage (**E**). Images of the bacterial community attached to the surface of the gill lamellae: Conventional SEM (**E**) and TEM—black arrows indicate the prokaryotic cells (**F**).

**Fig 3:**
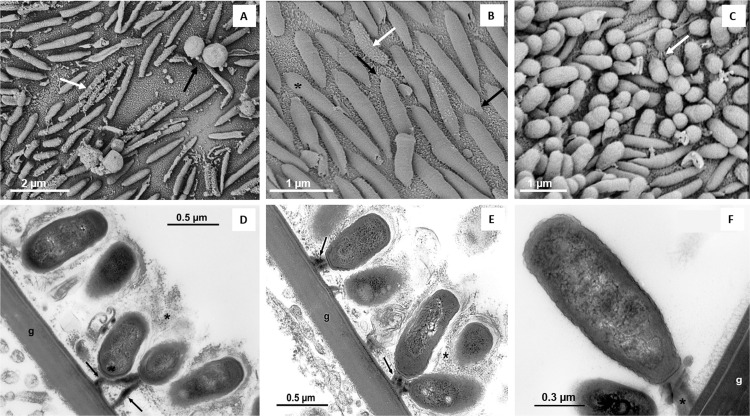
SEM images of bacteria associated with gills of *Cranuca inversa* (**A–C**) and TEM images of prokaryotes associated with gills of *Parisesarma guttatum* (**D–F**). Different morphologies are visible within the groups of prokaryotes on the gill surface: Rough (white arrow) and smooth horizontal rod-shaped, and cocci (black arrow) (**A**). Smooth and rough (white arrow) spindle-like prokaryotes with visible distal peduncles (black arrows) lying horizontally on the gill chitinous cuticle (**B**). Dividing prokaryotic cells (*). Prokaryotes perpendicularly attached to the gill cuticle (**C**). Filaments showing cell-gill attachment are visible (black arrow). Groups of prokaryotes near the gill (g) (**D**). Arrows = pili, arrowhead = large electron-dense filaments that extend from the terminal part of the prokaryotes towards the branchial cuticle. This image appears to indicate the mechanism of adhesion of the bacteria to the gills, while the pili are oriented on the opposite side of the gills. Groups of prokaryotes in contact with the gill (g) (**E**). The prokaryotes appear to be enveloped in a layer of amorphous material (*). Electron-dense filaments are evident, extending from the terminal part of the prokaryote towards the branchial cuticle (arrows). Detail of the large electron-dense filaments (*) which extend from the terminal part of the prokaryote towards the g, branchial cuticle (g) (**F**).

TEM and SEM examination revealed a dense layer of prokaryotic cells attached to the surface of the gill lamellae in all the 12 crab species analysed (Figs 1–3). Both SEM and cryo-SEM showed that gill structures were well-preserved and the lamellae were visible in all the crab species (Fig 2). Cryo-SEM revealed that prokaryotes were embedded in an extracellular matrix covering the entire gill surface (Fig 2A and 2B) that is visible because it covers the intra-lamellar space. Gill morphological details were clearly visible, such as dorsal spines (Fig 2A and 2B) and bulb-like structures in fiddler crabs (Gelasiminae) that prevent the lamellae from collapsing during air exposure (Fig 2B). SEM revealed a clear separation of the lamellae. The presence of prokaryotes was detected from a 35⨉ magnification as uneven, dark and rough patches on lamellae surfaces; lighter smooth patches, typically along the external edge of the lamellae, indicated areas without adherent cells (Fig 2D and 2E). A single layer of cells attached to the surface of lamellae was also identified at low magnification with TEM (Fig 2F).

SEM revealed two different prokaryote morphotypes: rod-shaped and spherical-shaped cells (Fig 3A). Most of the rod-shaped cells were positioned horizontally, flat against the lamella with spindle-shaped, distal peduncles (similar to prosthecae; Fig 3A and 3B) adhering to the surface gill cuticle; other rods were perpendicularly orientated with respect to the gill surface with more rounded cell extremities (Fig 3C). Some of the cells with perpendicular orientation were attached to the gill surface by visible wires or peduncles (Fig 3C), and TEM imaging revealed these anchoring structures to be electron-dense filaments (Fig 3D–3F). Replicating cells were detected (Fig 3B), indicating that the microbial layer is composed of active cells colonizing the gill surfaces. Most of the rod-shaped and spherical prokaryotes had smooth surfaces, but some showed rough surfaces with wires and spikes (Fig 3B; Table 2).

**Table 2:** Prokaryotic cell abundance on the gills’ surface of crab species.

	Total abundance			Relative abundance					
Species				Morphotype %		Rod appearance%		Rod adhesion mode %	
	Av prokaryotic cell/mm^2^			Rods	Cocci	Rough	Smooth	Horizontal	Perpendicular
All	1.82×10^6^	±	4.59×10^5^	98.10	1.11	0.06	98.04	84.69	13.41
*Thalamita crenata* (*N* = 10)	2.88×10^6^	±	8.27×10^5^	100.00	0.00	0.00	99.95	97.79	2.21
*Parasesarma catenatum* (*N* = 6)	1.59×10^6^	±	3.83×10^5^	100.00	0.00	0.00	100.00	100.00	0.00
*Parasesarma guttatum* (*N* = 10)	1.48×10^6^	±	6.20×10^5^	99.85	0.15	0.00	99.85	99.85	0.00
*Neosarmatium africanum* (*N* = 8)	1.39×10^6^	±	3.75×10^5^	99.92	0.08	0.00	99.92	99.92	0.00
*Metopograpsus oceanicus* (*N* = 8)	1.66×10^6^	±	3.72×10^5^	90.52	9.48	0.00	90.52	90.52	0.00
*Metopograpsus messor* (*N* = 8)	1.66×10^6^	±	5.07×10^5^	97.93	2.07	0.00	97.93	97.69	0.23
*Paraleptuca chlorophthalmus* (*N* = 10)	2.28×10^6^	±	5.12×10^5^	100.00	0.00	0.00	100.00	90.57	9.43
*Austruca albimana* (*N* = 8)	1.85×10^6^	±	3.21×10^5^	100.00	0.00	0.00	100.00	31.02	68.98
*Cranuca inversa* (*N* = 10)	1.96×10^6^	±	3.71×10^5^	99.61	0.39	0.59	99.02	96.65	2.95
*Ocypode saratan* (*N* = 8)	1.45×10^6^	±	3.42×10^5^	100.00	0.00	0.00	100.00	43.07	56.93

Total abundance of prokaryotic cells for all the species and for each species individually is reported as the average number of cells counted per mm^2^ of gill surface ± standard deviation. Relative abundances of different classes of cell morphotypes (rods/cocci), rod appearance (rough/smooth) and rod adhesion mode (horizontal/perpendicular) are reported on the total counts for each class. Number of specimen replicates are shown in the species column.

In all crab species, apart from *T*. *crenata*, there was no evident overlap of prokaryotic cells growing over the gills (Fig 1A–1N). In *Metopograpsus* species, *T*. *crenata* and *M*. *depressus*, prokaryotes were present in high densities without gaps between cells (Fig 1A–1D). In all the remaining species, prokaryotic cells were sparser with limited contacts between cells. Laying rods were mostly oriented with a parallel tendency one to the other, creating wave-like patterns on the visible gill chitinous surface. Prokaryotes with perpendicular attachment were denser, with less distance between cells (Fig 1A–1L).

**Fig 4:**
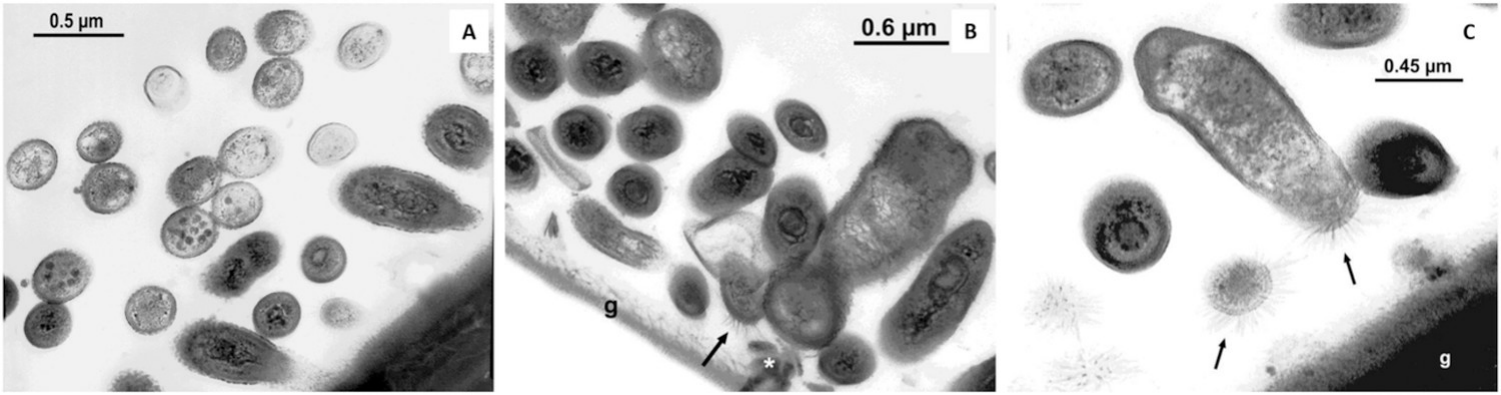
Prokaryotes morphology on the outer surface of the gill lamellae in *Tubuca urvillei*. Gill lamellae surface is indicated by ‘g’. Dense multilayers of bacteria are observed on the gill lamellae surface (**A, B**). The presence of pili and electron-dense filaments from the prokaryote cells to the gill lamellae surface is indicated by the arrow (**B, C**).

Multiple layer structures and abundant extracellular polymeric substances (EPS) embedding of the cells, typical of mature biofilms, were not detected by SEM, but nanowires, small prokaryote aggregates and sand debris were observed (Fig 1A–1L). On certain gill lamellae, layers of two-three prokaryote cells enveloped in a layer of electron-dense amorphous material were observed using TEM imaging (Figs 3D, 3E, 4A and 4B). Some of these cells showed a polarized morphology with pili oriented both towards and opposite to the gill surface (Figs 3D, 4B and 4C).

### Prokaryote quantification on the crab gill lamellae

**Fig 5:**
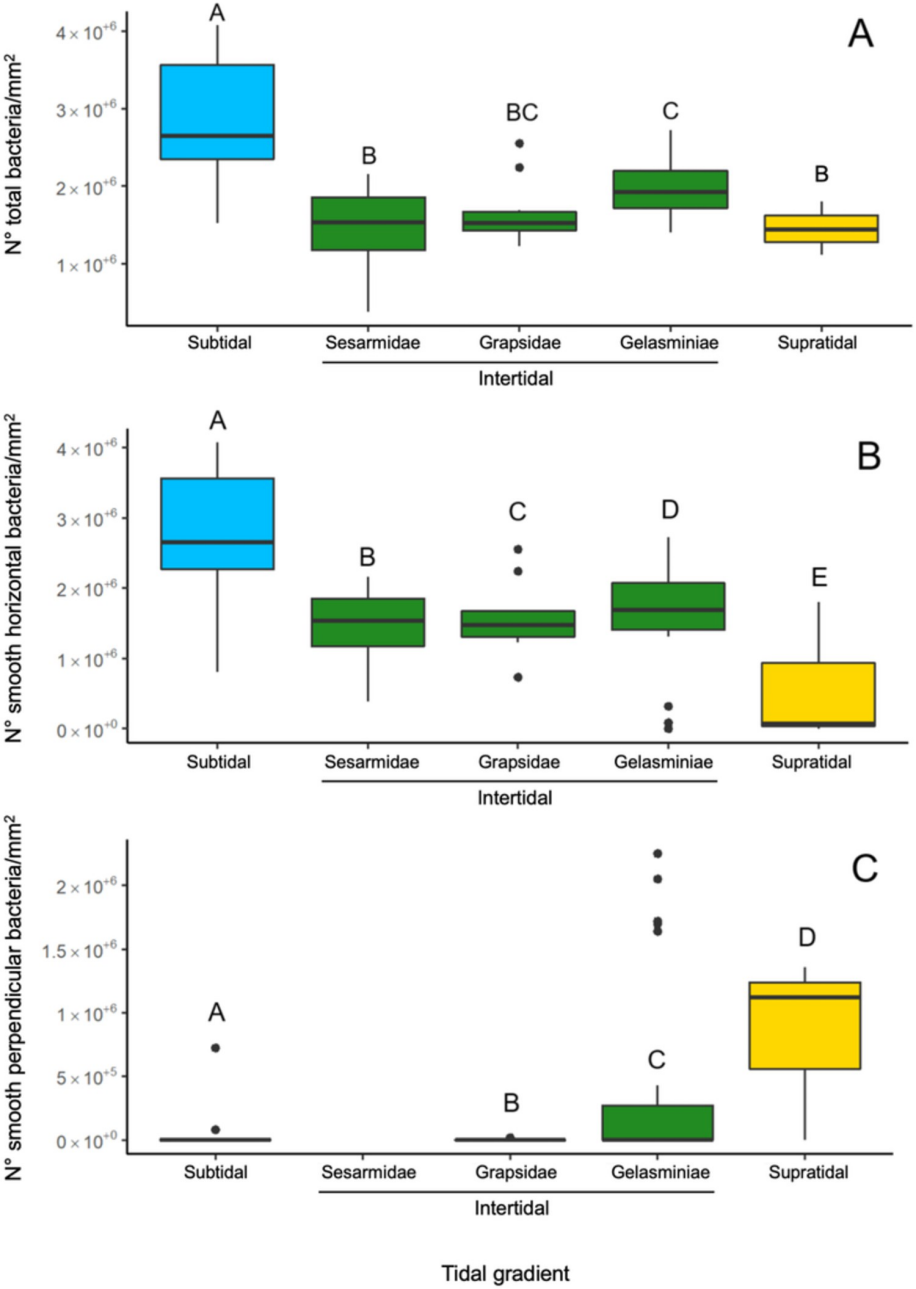
Prokaryote abundance computed through SEM imaging. Bacterial abundance on gills of crabs with different lifestyles along the tidal gradient. Letters are related to pairwise comparisons (**A**). Abundance of smooth horizontal rod-shaped prokaryotes across different intertidal levels. Letters show statistical significance from multiple comparisons (**B**). Abundance of smooth perpendicular rod-shaped prokaryotes across different intertidal levels (**C**). Letters indicate statistical significance among the groups resulting from multiple pairwise comparisons. The colours indicate the intertidal level: Blue = subtidal, green = intertidal, yellow = supratidal.

The abundance of prokaryotes on gill lamellae determined by analysis of SEM images differed according to the intertidal level inhabited by crab species (ANOVA: F_4,81_ = 12.54, *p* < 0.05; Multivariate GLM: Deviance_4,81_ = 41.8, *p* < 0.0001; Table 2, Fig 5A). Specifically, a significant difference between supratidal and subtidal species was observed with a trend of increasing abundance of prokaryotes from supratidal to subtidal species, with no significant difference among intertidal species and supratidal species (Table 2, Fig 5A). Analysis of the morphotypes revealed that rod-shaped cells were the most abundant across all the species (90%–100% of analysed prokaryotic cells, Table 2). Among these, smooth–rods were the most abundant (98.04% of rods), while rough rods appeared only in *C*. *inversa* (Fig 1G). Single cocci were found in four species (*P*. *guttatum*, *M*. *oceanicus*, *M*. *messor* and *C*. *inversa*) but in low abundance (1.11% of the total prokaryotes counted).

Focusing on the adhesion mode, horizontal rods comprised 84.69% of total prokaryotic cells counted on gill surfaces and were consistently detected in all crab species (Table 2). On the contrary, perpendicular rods were found in six species (*O*. *saratan*, *C*. *inversa*, *A*. *albimana*, *P*. *chlorophthalmus*, *P*. *catenatus* and *T*. *crenata*), comprising 13.41% of total prokaryotes counted, and were the main component on *A*. *albimana* (68.98%) and *O*. *saratan* (56.93%) gills.

A significant effect of crab habitat was observed for the abundance of smooth horizontal rod-shaped prokaryotic cells, with a consistent increase from supratidal to subtidal species (GLM; Deviance_4,81_ = 39961388, *p* < 0.0001; post-hoc analysis on figure caption; Fig 5B). Smooth perpendicular cells followed the opposite trend (GLM: Deviance_4,81_ = 38652872, *p* < 0.0001; post-hoc analysis in Fig 5C), with a significant increase from subtidal to supratidal species (i.e., *O*. *saratan*).

### Bacterial quantification by qPCR

Eubacterial DNA was successfully amplified in all the five sub-sampled species analysed using bacterial 16S rRNA gene quantification. A progressive decrease in bacterial abundance was observed along the tidal gradient, from the subtidal species *T*. *crenata* (3.11 × 10^6^ ± 2.31 × 10^6^ prokaryotic cells/mg gills) to the intertidal species (1.68 × 10^6^ ± 9.74 × 10^5^, 9.61 × 10^5^ ± 6.45 × 10^5^, 9.38 × 10^5^ ± 7.17 × 10^5^ for *M*. *messor*, *A*. *albimana* and *C*. *inversa*, respectively), reaching the lowest abundance in the supratidal species *O*. *saratan* (4.17 × 10^5^ ± 3.71 × 10^5^). The qPCR results differed among the five species (ANOVA: F_4,48_ = 9.798, *p* < 0.05): *T*. *crenata* was significantly different from *A*. *albimana*, *C*. *inversa* and *O*. *saratan* (Tukey’s multiple comparison tests, *p* < 0.05), while the three latter species were similar to each other (*p* > 0.05). *M*. *messor* significantly differed from *O*. *saratan* (*p* < 0.05) but not from *T*. *crenata* (*p* > 0.05).

## Discussion

In this study, we showed that the gills of mangrove crabs dwelling in the tidal area are consistently colonized by a dense layer of prokaryotes, with differential abundance and morphology according to the tidal level inhabited by the type of crab.

The specific micro-environment of the crab gill surface can represent a highly selective ecological niche for microorganisms. Gills are a constant source of nutrients [20]; in particular, nitrogen catabolites, excreted as ammonium ions, can attract and be used by ammonia-oxidizing prokaryotes, followed by denitrifying bacteria. As described in fishes, these bacteria can contribute to the detoxification of the host gill chamber by producing dinitrogen gas [50].

Bacteria are able to shift their morphology and adhesion mode in response to environmental triggers, such as nutrient source availability or changes in chemico-physical conditions [32,33]. For example, the presence and development of the stalk protoplasmic extension in *Caulobacter crescentus* is stimulated by the availability of environmental phosphate; the elongated stalk enhances the nutrient intake rate through the expression of specific membrane carriers [32,51]. Planktonic cells of *Pseudomonas aeruginosa* increase their adhesion capability according to the shear force intensity of the liquid medium they grow in [52]. The different microbial adhesion modes and morphologies detected in the present study may be selected by environmental stimuli and, thus, represent specific adaptations to the host eco-physiology along the tidal gradient.

Prokaryotes were found directly attached to the chitinous cuticle of the gill surface in all crab species apart from the subtidal species *T*. *crenata*, in which two layers of prokaryotes were observed (Fig 1A). High-resolution imaging also revealed the presence of thick electron-dense peduncles attaching the prokaryotic cells to the gill surface. These structures might have similar functions as nanotubes and intracellular cytoplasmatic bridges: interspecific communication structures (widely present across bacterial species, *e*.*g*., *Escherichia coli*, *Staphylococcus aureus* and *Bacillus subtilis*) for molecule exchanges [53,54]. Although such functional bridges have also been described in eukaryotic cell cultures (i.e., rat immune cells, [55]), no evidence of molecular communication in inter-kingdom structures has been reported [56].

To cope with the 30-fold higher concentration of oxygen present in the air compared to water, terrestrial animals evolved respiratory structures to reduce oxygen partial pressure and minimize oxidative damage during the gas exchange [57]. Tidal brachyuran crabs considered in the present study do not have complex structures, such as tracheae or lungs (i.e., those developed in truly terrestrial crabs such as *Pseudothelphusa garmani* and *Geocarcinus natalis*; [20]) to gate oxygen. Therefore, our results lead us to hypothesize that the prokaryotes present on the gill and their associated EPS, particularly at the high densities observed, may act as a physical barrier to reduce the oxygen partial pressure across the lamellar surface. Moreover, prokaryotes may have a chemical antioxidative function, consuming oxygen to sustain their metabolic pathways and producing antioxidant compounds [20,58,59]. Mangrove sediments are rich in hydrogen sulphide, which can adversely affect respiration enzymes and transport proteins. For this reason, a high number of marine invertebrates dwelling in mangrove sediments and shallow waters developed specialized chemo-symbiotic relationships with sulphur-oxidizing bacteria present on their gills (*e*.*g*., lucinid bivalves, [22]).

**Fig 6:**
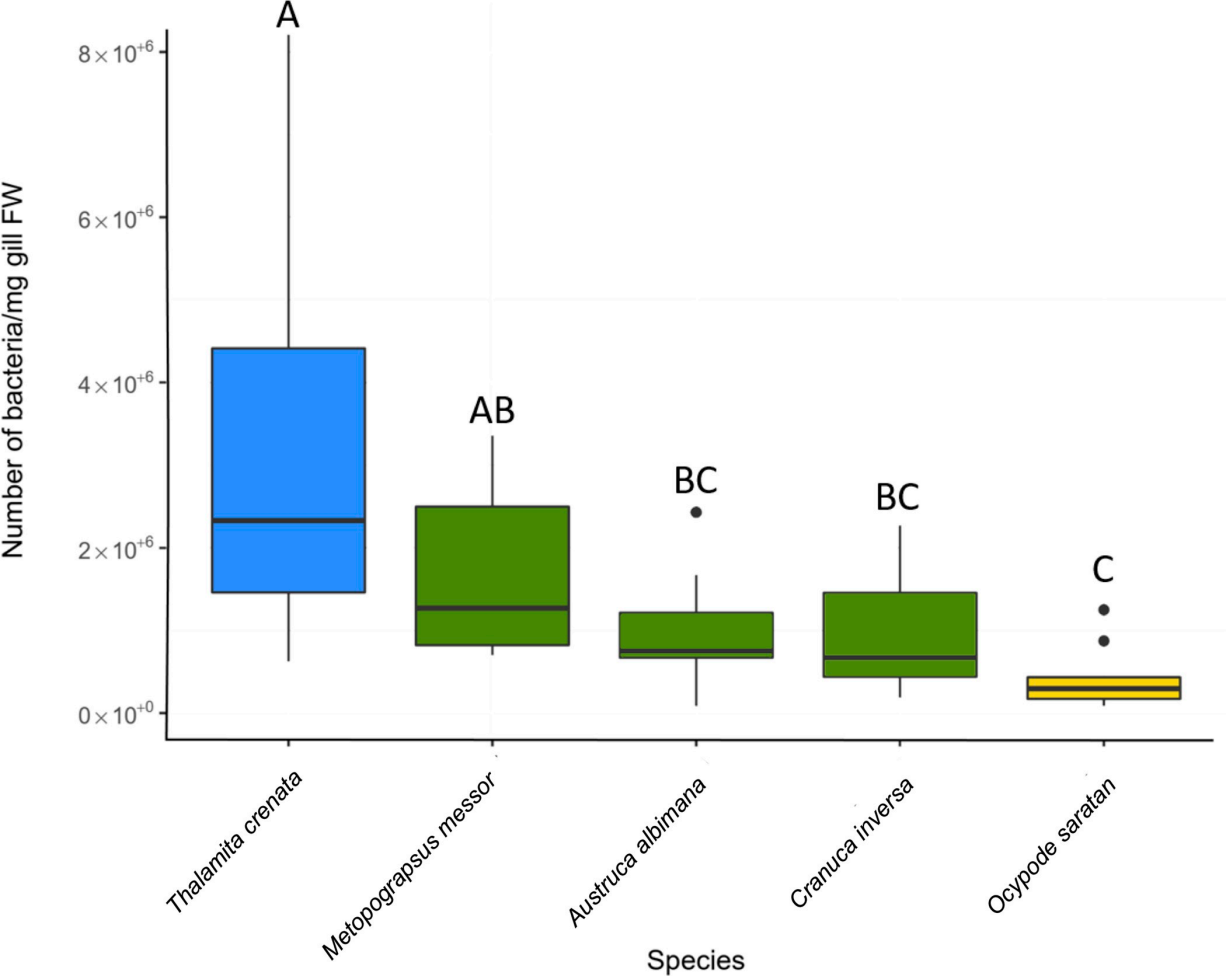
Bacterial quantification using qPCR analysis. Bacterial abundance on gills of five different mangrove crab species with different lifestyles along the tidal gradient. Letters indicate statistical significance (*p* < 0.05) among the groups resulting from multiple pairwise comparisons. The colours indicate the intertidal levels: Blue = subtidal, green = intertidal, yellow = supratidal.

Counts of prokaryotic cells using SEM imaging, showed a decreasing trend of cell density in the more landward-adapted species (Fig 5). qPCR analysis (Fig 6) corroborates (i) the SEM counting indicating that most of the prokaryotic cells imaged by SEM may belong to the bacteria Domain, and (ii) the significant decrease of bacterial numbers toward the supratidal species. The higher desiccation risk linked to emersion events, coupled with the high concentration of salts and catabolites accumulating on the gill surface [20], represents a strong selective pressure for marine prokaryotes, such as those present in the supratidal habitats. The two different adhesion modes observed with electron microscopy (i.e., perpendicular and horizontal rods), differed along the host tidal gradient: while horizontal rods were significantly more abundant in the subtidal crab species, perpendicular rods were more abundant in intertidal and supratidal species (Fig 5B and 5C). Although the detailed hydrodynamic of water streams through and around the gill lamellae have never been fully described, it is known that maxilliped flabellum and scaphognathite appendages represent the main generators of shearing forces inside the crab gill chamber [60–62].Flabella, continuously cleaning the lamellae from sediment and epi-parasites, can exert an important selective pressure on microbes colonizing the gill surface [62]; only those able to adapt their morphology and adhesion mode to such a mechanical challenge can develop a more stable association with the host. In our study, the high overall abundance of horizontal rod cells can represent an adaptation to such a selection: with a higher adhering surface compared to other morphologies and adhesion modes, they are likely to be more resistant to the maxilliped flabella action. The adhesion of prokaryotic cells to a surface can also be positively correlated with the stream intensity of the surrounding liquid medium [51], so the different content of water in the gill chamber can also be a determinant in selecting microbes. This process can play a role in the increasing abundance of bacterial colonization we observed in tidal crab gills along the land-sea gradient. In subtidal species the direct uptake of marine water has been described [60,61]; thus, the direct activity of flabella, coupled with the scaphognathite, can produce water streams around the gill lamellae. On the contrary, in intertidal and supratidal species, like *A*. *Albimana* and *O*. *saratan*, gills are moistened by absorbing water from the waterlogged sediment [63], leading to less intense shear forces on the gill surface.

## Conclusions

The results of this study suggest that prokaryotes, and in particular eubacteria, are a consistent component of the crab gill microenvironment across the different habitats created by the tidal gradient. Our findings also demonstrate that morphological patterns of prokaryote communities colonizing the gill surface change in accordance with the level of tidal adaptation of their host, providing the basis to further investigate the functional role of microbial communities in the terrestrialisation process in arthropods.

## Supporting information

S1 Data (XLSX)
